# The unstable total hip replacement

**DOI:** 10.4103/0019-5413.39667

**Published:** 2008

**Authors:** F D'Angelo, L Murena, G Zatti, P Cherubino

**Affiliations:** Department of Orthopedics and Traumatology, University of Insubria, Varese - Italy

**Keywords:** Dislocation, hip arthroplasty, revision

## Abstract

**Background:**

Dislocation is one of the most common complications of total hip arthroplasty with a reported dislocation rate of 3.2%. Despite increased experience with hip replacement, the overall rate has not yet changed. The aim of this paper is to review the most recent literature published on this topic and indexed in Medline, in order to clarify the main risk factors, and to standardize a treatment protocol of such an important complication of prosthetic surgery.

**Materials and Methods:**

Medline database was searched using key words: “hip dislocation”, “hip instability” from 1980-2007. Studies were eligible for review and included if they met the following criteria: (1) publication in English, (2) clinical trials (3) review papers.

**Results:**

The risk of first-time dislocation as a function of time after the surgery is not well understood. Most, but not all, series have demonstrated that the risk of dislocation is highest during the first few months after hip arthroplasty; however, first-time late dislocation can also occur many years after the procedure. Several risk factors were described, including the surgical approach, the diameter of the head, impingement, component malposition, insufficient abductor musculature. In addition, there are also many treatment options, such as long-term bracing after closed reduction, component reorientation, capsulorraphy, trochanteric advancement, increasing offset, exchange of the modular head and the polyethylene liner, insertion of constrained liner.

**Conclusion:**

Preventing hip dislocation is obviously the best strategy. Surgeons must take into account patient and surgical risk factors. For patients at high risk for dislocation the surgeon should accurately restore leg length and femoral offset; the use of larger femoral heads, posterior transosseous repair of the capsulotendinous envelope if posterior approach is chosen or the use of a lateral approach should be considered. Proper patient education and postoperative care are very important.

## INTRODUCTION

Total hip arthroplasty (THA) is extremely successful in alleviating pain and treating limitations of function as a result of coxarthritis.^1^,^2^ Unfortunately, hip instability is still one of the most problematic complications and continues to be one of the most frequent causes of early revision.^3^,^4^ Dislocation after THA causes an apprehensive and unsatisfied patient, a frustrated surgeon and could lead to medical malpractice litigation and high extra costs for the healthcare system. In a recent study it has been reported that the hospital cost of each closed reduction episode represented 19% of the hospital cost of an uncomplicated total hip arthroplasty and when revision surgery was needed this percentage rose to 148%.^5^

A complete understanding of the factors that play a role in the etiology of instability, techniques to reduce its incidence and a clear knowledge of treatment options are mandatory for surgeons that perform THA in their clinical practice. This review article reports about the etiology of hip arthroplasty dislocation and describes some methods for the management of this complication.

## MATERIALS AND METHODS

We did a computerized literature search using Medline to identify all citations concerning prosthetic hip procedures published from 1980 to June 2007 using the Medical Subject Heading terms “hip dislocation”, and “hip instability”. Studies were eligible for review and included if they met the following criteria: (1) publication in English, (2) clinical trials (3) review papers. The searches were supplemented with manual searches of bibliographies of the published articles and major orthopedics textbooks.

The articles were analyzed in line with research questions: definition of the problem, incidence, risk factors, clinical approach, treatment options. Papers focusing on late instability due to septic or aseptic loosening of implants were excluded as in these patients the primary pathology is the loosening and not the instability.

The definition of THA dislocation is complete loss of contact between the femoral head and acetabular component that requires intervention for reduction. Subluxation is usually considered a transient and incomplete loss of contact that self-reduces. Dislocation may occur early or late. Early dislocation occurs in the first three months postoperatively and has a lower rate of recurrence with better prognosis.^6^ Late dislocation can occur after several years of implantation and could be considered as a distinct pathologic entity with multifactorial etiology including polyethylene wear, soft-tissue laxity, neurological decline and eventually trauma.^7^ Dislocation can be single or recurrent: more than two dislocations are defined as recurrent.^8^

## DISCUSSION

### Incidence

Usually the risk of dislocation is identified between 1% and 3%. The Mayo Clinic database report a dislocation rate of 3.2% in 10,500 THAs. This rate is frequently cited because of the large sample size.^6^ The incidence of dislocation after THA varies greatly in orthopedic literature. It has been reported to range from less then 1% to more then 9%: rates vary depending on variables like size of study, the number and experience of surgeons involved and length of follow-up.^4^,^9^–^12^ Especially, follow-up duration is very important because the risk of dislocation doesn't seems to be constant but cumulative, increasing with time.^13^ Berry *et al.*, reported for Charnley hip replacement a cumulative risk of 1% at one month postoperatively, 1.9% at one year postoperatively, a constant 1% increase every five years, and a 7% increase at 25 years, affirming that the cumulative long-term risk of dislocation is greater than has been reported in short-term studies.^14^

The risk of dislocation is much higher after revision THA with ranges from 5% to 20%, depending on the series studied; this is to be addressed to the extent of soft tissue dissection and the complexity of this kind of surgery.^15^–^18^

## Risk factors

### Patients' factors

Ekelund *et al.*, reported that age was related to dislocation.^1^ Morrey *et al.*, showed that THA in patients older than 80 years had a twofold to threefold increase in the rate of dislocation compared with a younger group of patients but not all reports found that correlation.^19^,^20^ Other studies reported that elderly patients had a 4.5 times greater risk of dislocation.^21^ Berry *et al.*, in their paper on the cumulative long-term risk, affirm that the relative risk for dislocation in patients older than 70 years is 1.3%.^14^ In a recent study, based upon the Kaiser Permanente Total Joint Registry database for total hip arthroplasty procedures performed in Southern California, no increased risk in dislocation rate with advancing age was found: previously reported 9.2% dislocation rate in patients over 80 years of age reduced to only 3.7% when excluding the diagnosis of proximal femur fracture.^22^

Many studies have suggested that women are more likely to have a dislocation when compared to men.^6^,^13^,^14^,^23^ This could be related to increased preoperative range of motion (ROM) and a difference in tissue laxity. However, this is not supported by other authors.^24^,^25^ It has to be considered that the duration of follow-up may affect outcome because women live longer.

Body weight doesn't seem to be a risk factor for dislocation. Obesity, due to excess of adipose tissue, is related to a decreased ROM. On the other hand, a tall patient may be at risk because of the longer lever arm of the legs that generates a relative ease of translation of femoral head.^19^,^23^,^25^

Prior surgery such as osteotomy or conversion of a prior arthrodesis is strongly associated with increased rates of instability.^6^,^25^,^26^ Factors like compromised abductor function, bone loss, bone deformity could be the reasons of the increased risk associated with these conditions.

Developmental hip dysplasia is associated with increased rates of instability because of abnormal bone anatomy and altered muscle function.^27^ If a subtrochanteric osteotomy is performed the incidence of dislocation is reportedly higher at 14% (three of 21 patients).^28^

Other identifiable risk factors include history of fractures, osteonecrosis, or inflammatory arthropathy.^14^,^29^ Patients with Rheumatoid arthritis (RA) undergoing primary THA were found to have a higher risk of dislocation, probably because of impairment of other joints and inferior quality soft tissue. Berry *et al.*, recently reported a relative risk of 1.4 for patients with inflammatory arthritis as compared with osteoarthritis.^30^

A high American Association of Anesthesiologists (ASA) score was associated with an increased risk of dislocation.^31^ Cognitive dysfunction due to dementia, psychosis or alcoholism are reported as risk factors for hip instability.^27^,^32^–^33^ Cerebral palsy, polio, spinal cord injuries and all conditions that generate neuromuscular impairment can significantly place patients at higher risk for dislocation, affecting muscle tone and proprioception.^23^

### Surgical factors

Woo *et al.*, suggested that dislocation could be related to the volume of surgery performed by the surgeon.^6^ Surgeons who perform less than 10 THAs a year seems to have three times the risk of dislocation in the THA recipient according to some authors.^34^ A review of Medicare patients in the United States described varying rates of dislocation according to surgeon volume (1-5/y, 4.2%; 6-10, 3.4%; 11-25, 2.6%; 26-50, 2.4%; > 50, 1.5%).^35^ A recent study has confirmed that there is an obvious increase in dislocation rate in surgeons who perform less than 10 hip arthroplasties per year.^8^ A systemic review of the literature published in 2006 demonstrates a substantial positive association between surgical volumes and improvement in most THA outcomes, including dislocation; lower dislocation rates are associated with increasing surgical volume and this correlation appears to be stronger for surgeon volumes than for hospital volumes.^36^

Surgical approach is probably one of the most controversial factors that influence hip stability after THA. Woo and Morrey reported a dislocation rate of 5.8% for the posterior approach and only 2.3% for anterolateral approach.^6^ Masonis and Bourne reviewed 13203 primary THA in a large meta-analysis reporting a comprehensive review that included dislocation rates associated with surgical approach. They found dislocation rates of 1.27% for transtrochanteric approach, 2.18% for the anterolateral approach, 0.55% for the direct lateral approach and 3.23% for the posterior approach. The dislocation rates for the posterior approach with and without capsular repair were 2.03% and 3.95%, respectively.^37^ Woo and Morrey suggested that this increased incidence of dislocation with the posterolateral approach is caused by taking down the short external rotators and the posterolateral capsule.^6^ Capsular resection required with any approach other than posterior/posterolateral is usually less extensive.

As a consequence many authors suggested that a meticulous posterior capsular repair could decrease the dislocation rates to comparable levels to other approaches. Pellici *et al.*, reported a reduction from 4.1% to 0% in 395 patients with posterior capsular repair.^38^ Goldstein *et al.* obtained good results with capsulorraphy significantly reducing dislocation rate from 4.8% to 0.7% in a population of 1515 patients.^39^ White *et al.*, reported a reduction from 2.8% to 0.6% in 1000 patients.^40^ Laboratory studies support this theory. In a cadaver model, Sioen *et al.*, demonstrated that posterior transosseous repair of the capsulotendinous envelope resulted in better restoration of normal anatomy and increased stability without compromising movement.^41^ In a similar study, Mihalko and Whiteside demonstrated greater joint stability with repair of the capsule and external rotators.^42^ However, some authors questioned the benefit of repairing the posterior structures, stating that their integrity appeared disrupted at follow-up examination. Some studies using radiopaque markers measured on plain radiographs reported failure rates of between 63% and 80%.^43^,^44^ More recent studies using ultrasonography and radiostereometric analysis to assess the integrity of the posterior repair after THA showed that the short external rotators and capsule were intact in 89-100% of patients at three months postoperatively.^45^,^46^ In a recent meta-analysis on surgical approach Kwon *et al.*, found that posterior approach without soft tissue repair had 8.21 times greater relative risk of dislocation than with soft tissue repair. The same authors reported comparable dislocation rates associated with the anterolateral, direct lateral, and posterior approaches with soft tissue repair (0.70%, 0.43%, and 1.01%, respectively).^47^ Minimal incision THA has been associated with an increased complication rate, but Lorio *et al.*, recently reported that a 10 cm mini incision posterior approach with enhanced posterior soft tissue repairs presented a dislocation rate comparable to a standard access (1.7% and 1.3% respectively) and lower than 5.5% dislocation rate reported in a group of patients in which soft tissue repair wasn't done.^48^

Component orientation is a very important factor affecting the stability of an implant and also plays an important role in the long-term success influencing the production of wear debris. Femoral component positioning should be done in 15° anteversion, but usually inadequate version and excessive abduction on the acetabular side are the two most frequent errors that lead to instability. Acetabular position, in fact, is not always so reproducible during common clinical practice. Hendrix *et al.*, studied a group of patients who had THA and showed that although the mean acetabular abduction angle of 42° was close to the standard target of 40°, mean acetabular abduction ranged from 22° to 57.2°. This variation could be due to variations in patient position and pelvic movement during surgery and it could probably be reduced by navigation.^49^ Lewinnek *et al.*, have reported on a “safe zone” for cup orientation; anterversion 15° ± 10° and abduction 40° ± 10°. Higher dislocation rates were seen when the cup position was outside of this zone.^50^

Others risk factors for dislocation are nonunion of greater trochanter and the lack of recognition of impinging soft tissues and/or osteophytes.

Implant design has an important role on hip stability. Size of femoral head, shape and size of neck, femoral offset, head-to-neck ratio and socket depth should be considered when choosing an implant for any patient. Inadequate offset and length on the femoral side have proven to be causes of instability.^51^ Failure to achieve adequate length leads to a decrease in myofascial tension and raises the risk of dislocation. On the other hand if lateral femoral offset is reduced there is a reduction of the lever arm of abductor muscles with consequent decrease in myofascial tension. With inadequate femoral offset early impingement of the proximal femur against the pelvis can occur and this could be another source of instability.

A larger femoral head is an attractive choice when stability is a critical factor. In a recent report on a consecutive series of 254 primary hip arthroplasties performed by a single surgeon Padgett *et al.*, reported a 4.8% rate of dislocation at a minimum followup of 2 year. Stratified by head size, the dislocation rates were 3.6% for 28-mm, 4.8% for 26-mm, and 18.8% for 22-mm bearings.^52^ Patients who are at higher risk for dislocation will have a less likely chance of complications with a larger head. Larger femoral head diameters reduce the risk of dislocation because they improve the head-to-neck ratio, which increases the primary arc of motion before impingement. A greater amount of translation of femoral head is required before dislocation occurs, the head sitting deeper in the liner. Larger heads allow for greater neck length without the use of the skirted necks that can be a source of component impingement. Finally, there may be greater soft-tissue resistance to dislocation as the femoral head is better contained by the surrounding soft-tissue envelope.^53^,^54^ Berry *et al.*, recently reported that in THA, a larger femoral head diameter was associated with a lower long-term cumulative risk of dislocation. The femoral head diameter had an effect in association with all operative approaches, but the effect was greatest in association with the posterolateral approach.^55^ In the past long-term concern about volumetric wear associated with the use of large femoral head has led surgeons to prefer 28-mm head. Advances in tribology and the advent of highly cross-linked polyethylene now permit the safe use of thinner polyethylene inserts and larger femoral head. Total hip arthroplasty with large femoral heads articulating with a highly cross-linked polyethylene showed excellent wear characteristics and clinical results in the short-term period.^56^

Another implant augment designed to reduce the rate of dislocation is the elevated liner on the acetabulum. Its efficacy has been shown but it must be considered that the elevation improves stability in only one direction while it could be a source of impingement causing the head to lever out in the opposite direction.^57^

### Clinical approach to the unstable hip

When approaching an unstable hip it's important to determine the time and direction of instability. For early instability it's possible that abductor motor function is not yet recovered and that capsular healing isn't yet complete. Late instability is generally associated with other variables including polyethylene wear, soft-tissue laxity, neurological decline. The direction of instability is related to surgical approach and it is important information when considering treatment options. Early anterior dislocation associated with a posterior approach can be usually treated conservatively with good results avoiding external rotation and extension.^58^ On the contrary early posterior dislocation associated with posterior approach is more likely to recur in the future. How the hip was reduced and ease of reduction, safe ROM, the number and frequency of dislocations should be well investigated when collecting clinical history.

Observation of gait, abductor weakness, pelvic dropping should be considered during examination of patients. The hip wound should be checked considering type of incision and eventual erythema around it. Leg length should be measured from the anterior superior iliac spine to the medial malleolus. Strength of muscles around the hip should be assessed: active straight leg raising and side lying abduction are very accurate and easy tests. Range of motion should be tested actively and passively paying attention not to cause dislocation of the implant. The examination should be completed with a neurological evaluation of strength and sensation of the lower extremities.

An anteroposterior (AP) view of the pelvis and a true lateral view of the hip are the standard X-rays needed for a radiographic evaluation. As regards the acetabular shell the exact location should be understood considering distances from and Kohlers line. Version of the acetabular and femoral components should be observed. For the shell the inclination and abduction angle, considering any pelvic obliquity, must be noted. It's important to consider offset and to compare it with the contralateral hip. Finally the length of both legs should be observed measuring the distance of the lesser trochanter from the interteardrop line and the presence of heterotopic ossification. If more information on component orientation and position are needed in difficult cases computed tomography (CT) scan can be obtained.^59^

### Treatment

Management of the dislocated THA is difficult. If the components are well positioned, the hips can be usually relocated using closed reduction; no further dislocation occurs in about 67% of these patients.^6^ Closed reduction should be done in the presence of adequate sedation and muscle relaxation, eventually using intravenous sedation, general or regional anesthesia. Repetitive attempts of closed reduction can damage the femoral head as recently reported by Schuh *et al*.^60^ Safe ROM should be assessed by the surgeon immediately after reduction. If it is possible to reach at least 90° of flexion with 15° arc of internal and external rotation without sensation of instability, in the case of first episode of dislocation with radiographs showing acceptable position of implants, a conservative treatment should be attempted. The use of a brace is controversial. Mallory *et al.*, showed success rates greater than 70% in eliminating subsequent dislocation.^61^ Dewal *et al.*, found no difference between bracing and nonbracing in the prevention of recurrent instability in a group of 91 first-time dislocations and in another group of 58 recurrent dislocations.^62^ Optimal duration of brace treatment has not been investigated with randomized trials. However, the objective of brace is to protect pericapsular structures disrupted after dislocation till healing.^63^ Therefore brace should be used for six to 12 weeks, following this period with strict hip precautions for another eight weeks. For posterior dislocations, brace should limit flexion to 70°; for anterior dislocations that occur in extension and external rotation, hip extension should be eliminated and sometimes brace can be set for an extension lock at 30° hip flexion. Rotations are probably the more important movements that have to be controlled, therefore it is necessary to use a long-leg extension brace that incorporates the foot.

In case of malpositioned components, recurrent instability (more than two episodes), irreducible dislocation, inadequate soft-tissue tension and chronic dislocation an operative treatment should be programmed. Daly and Morrey reported that when a cause of instability can be identified and addressed to one prosthetic component, revision of that component has a successes rate of 70% in eliminating instability.^26^ If the components are malpositioned, they should be revised, having simple polyethylene exchange (PE) and the use of elevated or extended liners has a limited role in this cases. In a recent report on a series of patients with recurrent instability that required revision surgery, cup malpositioning was the major cause for 35% of patients; revision surgery was successful with no recurrence of instability in 91% of cases.^64^

Impingement or abductor deficiency can be the cause of dislocation in the presence of well-positioned components. Soft tissue tension is difficult to assess by clinical examination or by radiographs. Measurement of femoral offset, especially when compared to the contralateral hip or preoperative radiographs, can give some information on soft tissue tension. Loss of femoral offset of more than 1 cm should raise concern regarding myofascial tension of hip abductors. Impingement can be related to the implant characteristics or the patient's femoral and pelvic anatomy and it is usually assessed at the time of revision. For patients who do not have gross malpositioning of the components or abductor dysfunction problems, modular component exchange and debulking of offending soft-tissues and osteophytes can give satisfactory results. Using modularity of implant the surgeon can increase neck length, increase femoral head size, or use lipped, anteverted or lateralized acetabular polyethylene liners.

Treatment of a loss of myofascial tension from a decrease in offset or limb shortening can be difficult. Some authors advocate in these cases the use of trochanteric advancement.^1^,^23^ Nowadays this technique is rarely used. It was developed when monoblock implants were used and the surgeon did not have the options given now by modularity. It continues to be an option in the presence of proximal migration of a nonunited trochanter after a trochanteric osteotomy.^65^,^66^ Trochanteric advancement has the advantage of leaving the implant untouched, but if loss of offset presents as laxity associated with bony impingement resulting in dislocation then stem revision is mandatory.

Toomey *et al.*, reported with modular component exchange a success rate comparable with that of more extensive operations.^67^ Beaule *et al.*, reported on a series of patients treated with jumbo femoral heads for recurrent instability. At an average follow-up of 6.5 years, 84% cases had no further episodes of instability.^68^ The results of jumbo femoral head for revision in the presence of instability seem to be good but the size of the acetabular component and availability of suitable liners remains a drawback.

The use of bipolar hemiarthroplasty is an alternative to jumbo femoral head for the treatment of recurrent dislocation. The bipolar head usually is larger in diameter and provides additional stability. Parvizi and Morrey^69^ reported 81% success in terms of gained hip stability using this technique at an average follow-up of five years. Unfortunately, approximately half of the patients in their group had moderate hip symptoms and Harris Hip scores improved modestly from a mean of 24 to 55 points postoperatively. The problem of this technique is related to the bipolar articulation on cartilage denuded acetabular bone, resulting in hip pain and gradual erosion of the acetabular bone stock.

Constrained liners are a relatively recent treatment option for patients with recurrent instability that is gaining popularity among surgeons. The main indications for this technique are instability in the presence of cognitive or neuromuscular disorder and/or severe abductor deficiency, multiple failed revisions for instability without a constrained socket and unidentifiable cause of hip instability. Nevertheless constrained liners present the disadvantages of thin polyethylene and restricted range of motion that could result in impingement, osteolysis due to wear debris, early acetabular loosening or separation of individual components. This option should be considered as a salvage attempt for particular cases and should not be used when a correctable cause of dislocation is identified. Berend *et al.* recently reported an overall dislocation rate of 17.5% in a series of 755 consecutive constrained acetabular components. When considering the use of constrained socket for the treatment of recurrent hip instability the redislocation rate was 29%. Furthermore, the authors' had overall failure rate of 41% secondary to other causes like loosening, periprosthetic fractures, infection, and dissociation.^70^ Goetz *et al.*, evaluated 56 hips, with an average follow-up of 10 years, treated with a constrained component. Recurrent dislocation occurred in only 7% of cases. Overall failure rate was 21%.^71^ Some authors cemented a constrained liner into a well-positioned, well-fixed acetabular component reporting 94% success rate at a mean follow-up of 3.9 years.^72^

### CONCLUSIONS

Preventing hip dislocation is obviously the best strategy. Surgeons must take into account patient and surgical risk factors. For patients at high risk for dislocation the surgeon should accurately restore leg length and femoral offset; the use of larger femoral heads, posterior transosseous repair of the capsulotendinous envelope if posterior approach is chosen or the use of a lateral approach should be considered. Proper patient education and postoperative care are very important. First episode of dislocation can be treated with closed reduction techniques in most cases. Recurrent instability should be surgically treated. Clear understanding of underlying causes of instability is the key for a successful revision surgery. The use of larger femoral head size, lengthening the femoral neck or modifying the acetabular polyethylene liner could be treatment options in the presence of well-positioned components. If acetabular shell or femoral stem are malpositioned, revision of the component is the best choice. Revision with constrained acetabular components should be considered as salvage procedures when other conservative and surgical treatments have failed. Conversion of an unstable THA to bipolar hemiarthroplasty should be reserved for elderly low-demand patients.
